# Performance of Manufactured and Recycled Steel Fibres in Restraining Concrete Plastic Shrinkage Cracks

**DOI:** 10.3390/ma16020713

**Published:** 2023-01-11

**Authors:** Talal O. Alshammari, Kypros Pilakoutas, Maurizio Guadagnini

**Affiliations:** 1 Department of Civil and Structural Engineering, The University of Sheffield, Sir Frederick Mappin Building, Mappin Street, Sheffield S13JD, UK; 2 Department of Civil and Structural Engineering, College of Engineering, Jouf University, Sakaka 72388, Saudi Arabia

**Keywords:** early-age concrete cracking, plastic shrinkage cracks, concrete shrinkage, recycled tyre steel fibre, manufactured steel fibres, steel-fibre-reinforced concrete

## Abstract

Early-age plastic shrinkage cracks can reduce the durability of concrete slabs by creating direct paths for the ingress of aggressive agents and thus accelerating degradation due to environmental attack, in particular, in hot and windy environments. The elimination of such cracks is essential for durable and sustainable concrete structures. This paper parametrically investigates the effect of manufactured steel fibres (MSF) and recycled tyre steel fibres (RTSF) on restraining plastic shrinkage and micro cracks at different dosages (10, 20, and 30 kg/m^3^). The plastic shrinkage tests were carried out in a specially designed chamber, according to ASTM C1579. Various environmental conditions are examined, and their impact on compressive strength and crack potential is assessed. A digital image analysis technique is used to measure length, width, and the area of the crack on the exposed surface to gain additional insights into crack behaviour. The results show a slight early-age (one-day) increase in compressive strength for the concrete exposed to the various environmental conditions, mostly as a result of higher temperatures. Through the use of the crack reduction ratio (CRR), both RTSF and MSF are shown to be successful in controlling plastic shrinkage and micro cracks, with the RTSF being superior due to the fact that they are better distributed in the concrete volume. The addition of 30 kg/m^3^ of RTSF was effective in preventing crack development in most environments or restraining cracks in extremely harsh environments. The adoption of these results will lead to more sustainable concrete slabs in the harsher environmental conditions created by climate change.

## 1. Introduction

Early-age cracking due to autogenous and plastic shrinkage is a key issue that affects concrete durability and reduces the lifespan of concrete structures. It is estimated that plastic shrinkage cracking is the source of roughly 80% of the early-age cracking in reinforced concrete structures [1,2]. Though plastic shrinkage cracks affect most structural members, they are more likely to occur in large surface area structures, such as slabs and pavements, and also walls [3].

Plastic shrinkage cracks are the result of volume changes that occur during the plastic stage, i.e., before the concrete hardens. These include air void expulsion and aggregate plastic settlement, as well as the bleeding and evaporation of water [4]. As the layer of bleeding water at the surface of concrete evaporates, water menisci develop between solid particles, causing the initiation of capillary pressure build-up. This capillary pressure has a limit and when this is reached, cracks develop [5]. Depending on the initial water content and the rate of evaporation, these cracks can be extensive and penetrate deep into the concrete surface. Cracks that initiate during the plastic state can progress until the final setting time and subsequently become drying shrinkage cracks [6,7,8,9]. Cracks facilitate the ingress of chemicals into concrete and accelerate concrete deterioration [10]. It is well accepted that such concrete cracks should be controlled and/or avoided.

Plastic shrinkage cracks occur due to rapid evaporation; hence, they are a direct consequence of environmental conditions, such as elevated temperature, low relative humidity, solar radiation, and exposure to high wind flows [11,12,13,14]. Such environments are encountered more often as a result of climate change and also as a result of population expansion and consequent exposure to more hostile environments, especially in dry/arid regions [15].

### 1.1. Plastic Shrinkage Phenomenon

**Figure 1:**
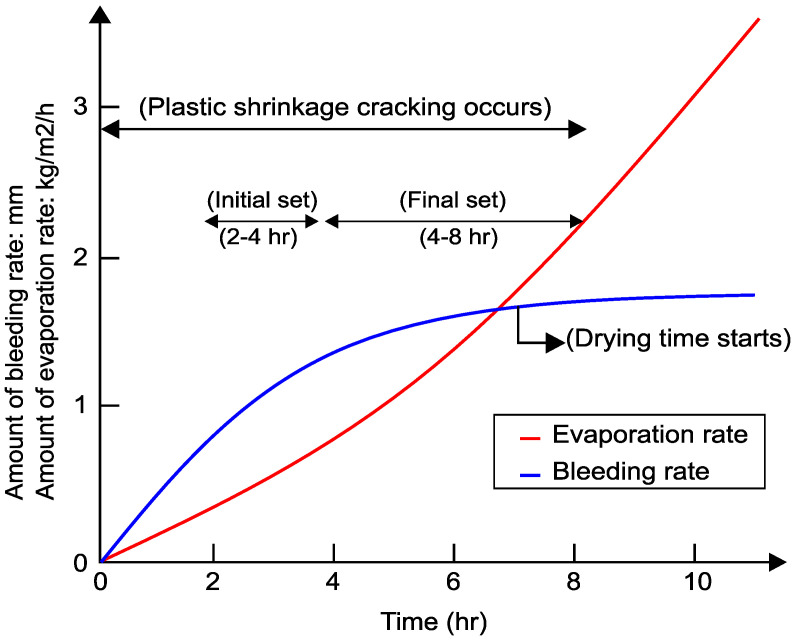
Evaporation and bleeding of concrete.

Plastic shrinkage cracking is expected to develop when the evaporation rate is greater than 1.0 kg/m^2^/h, which is most likely to occur in hot weather concreting in arid climates [16]. Kwak and Ha [17] investigated the relationship between bleeding and evaporation rate. Figure 1 shows a typical evolution of evaporation and bleeding rate and the likely period of plastic shrinkage.

Soon after casting and during the initial setting time (2–4 h) (see Figure 1), the denser solid constituent materials tend to sink and can become unable to hold onto excess mixing water. This water migrates towards the surface and some bleeds out of the mix onto the surface [18]. The rate of bleeding depends on the water content, particle size distribution, viscosity, and rate of hydration of cement.

After the initial setting time, the rate of bleeding water stabilises and then ceases. During the final set (4–8 h), the hydration of cement causes the surface temperature to increase, and, as a result, the evaporation rate can exceed the bleeding rate. A high rate of evaporation, also aggravated by dry conditions and wind, can lead to plastic shrinkage cracking before the final set ends [19].

**Figure 2:**
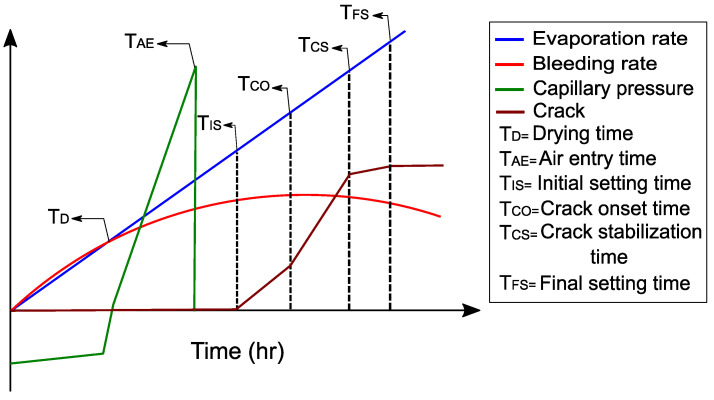
Typical behaviour of plastic shrinkage cracking.

Boshoff and Combrinck [20] and Sayahi et al. [21] studied the behaviour of plastic shrinkage cracks and found that the capillary pressure develops rapidly once drying begins but reduces suddenly when air enters into the pores, before the initial setting time begins. The development of capillary pressure is shown schematically in Figure 2 together with the typical phenomenological behaviour of plastic shrinkage [20]. When initial setting time (TIS) starts, bleeding is slowed down and can be considered to end by the final setting time (TFS). Soon after the initial setting time, the onset of cracking (TCO) begins, and the crack width increases up to the final setting time. When the final setting time is reached, the crack growth reduces. This subsequent crack growth is the result of temperature, autogenous shrinkage, and drying shrinkage.

### 1.2. Use of Fibres to Mitigate Shrinkage-Induced Cracking

Most recent research on plastic shrinkage cracks focuses on performance and durability enhancement of concrete as a material [22,23,24] and attempts to find solutions to avoid cracks or at least reduce their appearance. One of the most effective solutions to mitigate the shrinkage phenomenon is the use of different types of fibres in concrete, including steel, glass, polymer, and natural fibres.

Lee and Won [25] evaluated the effect of adding nano-synthetic fibres and hooked-end-type steel fibres (MSF) to concrete at volume fractions of 0.26%. The results showed that nano-synthetic fibres performed better than MSF to reduce crack width by about 36% but were not able to prevent the development of surface cracking. Mazzoli et al. [26] compared the ability of four types of fibres, including polypropylene (with three different lengths), polyvinyl alcohol, polyethylene, and steel (hooked ended), to control early-age shrinkage cracking (plastic and autogenous). The study concluded that polypropylene and polyethylene macro fibres were more effective at reducing the total crack area than MSF. Sivakumar and Santhanam [27] examined different combinations of fibres, such as MSF, polyester, polypropylene, and glass, and concluded that the combinations of MSF and polyester fibres had the best performance in controlling concrete plastic shrinkage cracking. This can be attributed to the fact that polymer fibres tend to have a smaller diameter and, as such, are better distributed.

Booya et al. [2] examined the effects of Kraft pulp fibres on plastic shrinkage cracking and found that these fibres are highly effective in controlling both micro and plastic shrinkage cracks. The use of natural fibres, such as flax and hemp, to restrain micro and plastic shrinkage cracks in concrete and mortar also compared well with polypropylene fibres [28]. Many other studies investigated different types of natural fibres to reduce plastic shrinkage cracks in concrete. Wang et al. [4], Balaguru [29], Soroushian and Ravanbakhsh [30], and Booya et al. [2] used natural cellulose fibres and found that they can reduce plastic crack widths but do not prevent concrete from cracking due to the high water absorption of the fibres compared to other types of fibres. Other studies examined the effects of sisal and coconut fibres to restrain plastic shrinkage cracks, and the results showed that sisal and coconut fibres can positively help reduce crack widths, but high volume fractions are needed to control plastic shrinkage cracks [31,32,33]. Nevertheless, natural fibres tend to reduce concrete strength, as well as increase permeability, and hence have limited general practical use in structural concrete. Hence, more robust and sustainable solutions are required.

For example, Karalar et al. [34] conducted a study that investigated the use of waste lathe scraps in reinforced concrete beams. The results showed that the addition of steel waste lathe scraps increased the compressive strength of the resulting concrete by up to 32.5% when a fibre volume of 3% was used and improved the beam’s mechanical performance. Moreover, Ali et al. [35] examined the effects of lathe waste scrap on both fresh and hardened properties of fibre-reinforced concrete and observed that while an increase in compressive and tensile strength can be observed at increasing fibre volume, workability is negatively affected.

With around 1.5 billion tyres worldwide being discarded every year, each containing about 10% of highly engineered steel cord as reinforcement [36], various research groups have focused on examining the suitability of recycled tyre steel fibres (RTSF) as a more sustainable alternative to manufactured steel fibres (MSF) [37,38]. Recent findings have shown that when steel cords are extracted from tyres, mainly via mechanical shredding, they can be further processed to remove impurities and long lengths, which can cause balling, and turned into effective fibre reinforcement [39,40,41,42,43].

Recycled tyre steel fibres (RTSF) are typically characterised by a range of lengths and a smaller diameter than conventional MSF and may offer a more holistic shrinkage crack control (from plastic to drying), as well as structural reinforcement benefits. Su et al. [44] tested the mechanical and drying shrinkage properties of mortars, including plastic rubber (PR) (2%, 5%, 7.5%, and 10% by volume) and RTSF at a constant volume fraction of 0.2%. The results showed that the addition of RTSF and PR had increased the compressive strength up to 14–27% and reduced the drying shrinkage crack lengths by about 25% at 7 days curing. Jafarifar et al. [45] studied the impact of adding RTSF (around 2.5% by weight) on the drying shrinkage of concrete pavements and found that the RTSF had the ability to control drying shrinkage compared to plain concrete. Graeff et al. [46] also found that the RTSF help in freeze–thaw, corrosion, and fatigue resistance. Al-musawi et al. [47] examined the effects of clean RTSF on the drying shrinkage of concrete made with different types of cement (CSA—calcium sulfoaluminate cement and RSC—calcium aluminate cement). The study showed that the inclusion of RTSF reduced the drying shrinkage strains by approximately 12%. Zhong and Zhang [48] examined the properties of concrete reinforced with three types of fibre, (i) manufactured steel fibre (MSF), (ii) recycled tyre steel fibre (RTSF), and (iii) polypropylene fibre (PPF). Surprisingly, the results showed that the fibres used did not significantly improve the mechanical properties in terms of compressive, splitting tensile, and flexural strengths compared to plain concrete. Nonetheless, the addition of RTSF had less impact on the workability of concrete than MSF and PPF and controlled drying shrinkage to a higher degree. Hence, although there is some uncertainty as to the effect of fibres on the overall performance of concrete, RTSF show real promise in enhancing shrinkage performance and thus warrants further studies.

### 1.3. Restrained Plastic Shrinkage Testing Techniques

As for all shrinkage cracks, plastic shrinkage cracks appear on the surface of the concrete as a result of external restraint. Hence, in plastic shrinkage tests, there is an attempt to amplify restraint, so as to accelerate cracking. Four main plastic shrinkage test techniques are used to evaluate plastic shrinkage cracks: ring, longitudinal, slab, and substrate restraint.

Bjøntegaard and Sellevold [49] adopted the ring test to restrain plastic shrinkage cracks in concrete. Concrete was placed between two concentric steel rings to a depth of 45–50 mm. The rings rest on a rigid plate and have 12 rigid ribs to restrain plastic shrinkage. The wind speed was controlled above the concrete specimens to achieve faster than normal drying time. The plastic shrinkage cracks occurred around the ribs. Ling et al. [50] used this arrangement in a more recent study and found that the ring test achieved high levels of plastic shrinkage cracks.

A test method relying on prisms with a size of 40 × 40 × 500 mm (longitudinal test) was first proposed in [51]. The arrangement utilises two bars at the two ends of the beams to restrain plastic shrinkage cracks. The test was further developed by Mora-Ruacho et al. [52] using larger specimens (150 × 150 × 600 mm) and including a riser in the centre of the specimen to increase plastic shrinkage cracking potential and localise the cracks above the riser. These types of arrangements eventually led to slab tests and substrate restraint tests that are more representative of real slabs, such as the test adopted here, as recommended in ASTM C1579 [53] and described in detail in the following section. The careful control of environmental conditions is also key for such tests [54,55].

### 1.4. Measurements

Tracking plastic shrinkage cracks is challenging due to the random initiation time, location of crack, and irregular crack shape, which renders conventional strain measurements inadequate. Therefore, dynamic manual and image-based techniques are necessary. Manual methods include the use of microscopes and crack magnifiers to measure the crack width and length. It is accepted that it is necessary to obtain the measurements at more than one point and utilise the average of the measurements [3,55,56,57]. The advantages of these methods are that they are relatively simple and can be applied easily on site. On the other hand, these methods have been criticised due to the need for human judgement and are more prone to errors compared to other measurement techniques.

Image-based techniques include digital image correlation (DIC) and digital image processing (DIP). Those methods tilize high-resolution cameras that capture images of the entire concrete surface and these images are subsequently processed by specially developed algorithms. These methods can track the crack width, length, and area at the surface of the concrete from the time of casting to the end of the test. Recently, DIC has been used extensively in studies monitoring cracks on hardened concrete surfaces [36,58]. In this study, a digital image processing method will be used to measure the crack width, length, and area.

### 1.5. Significance of Research

This paper aims to examine the impact of RTSF on plastic shrinkage cracking of concrete. A direct comparison is made with MSF and the impact of fibre distribution on crack initiation and development is investigated. Crack measurements are obtained through the implementation of a digital image processing method, in an attempt to have a more in-depth understanding of crack development and remove subjectivity. The use of finer structural fibres may lead to more sustainable and durable steel-fibre-reinforced concrete (SFRC) structures.

## 2. Experimental Program

### 2.1. Materials

#### 2.1.1. Manufactured Steel Fibre (MSF)

**Figure 3:**
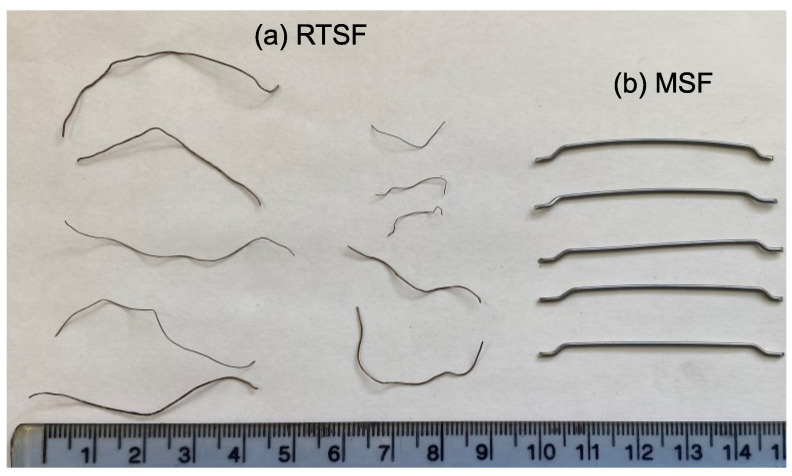
Appearance of (**a**) RTSF and (**b**) MSF.

In this study, hooked-end fibres (Figure 3) were selected as they are cost-effective and are commonly used in large flat-slab construction. The fibres have a length of 50 mm and a diameter of 1.0 mm, and a nominal tensile strength of 1150 Mpa.

#### 2.1.2. Recycled Tyre Steel Fibres (RTSF)

**Figure 4:**
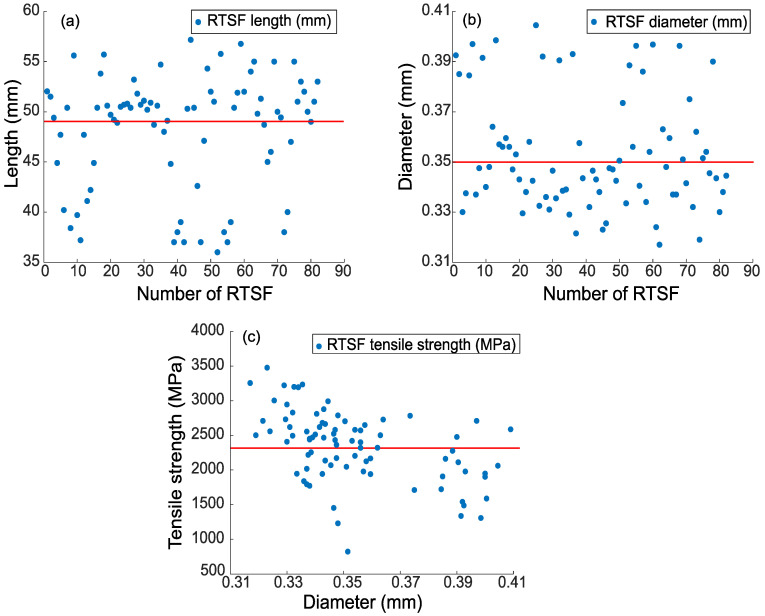
Average (**a**) length, (**b**) diameter, and (**c**) tensile strength of the RTSF.

**Figure 5:**
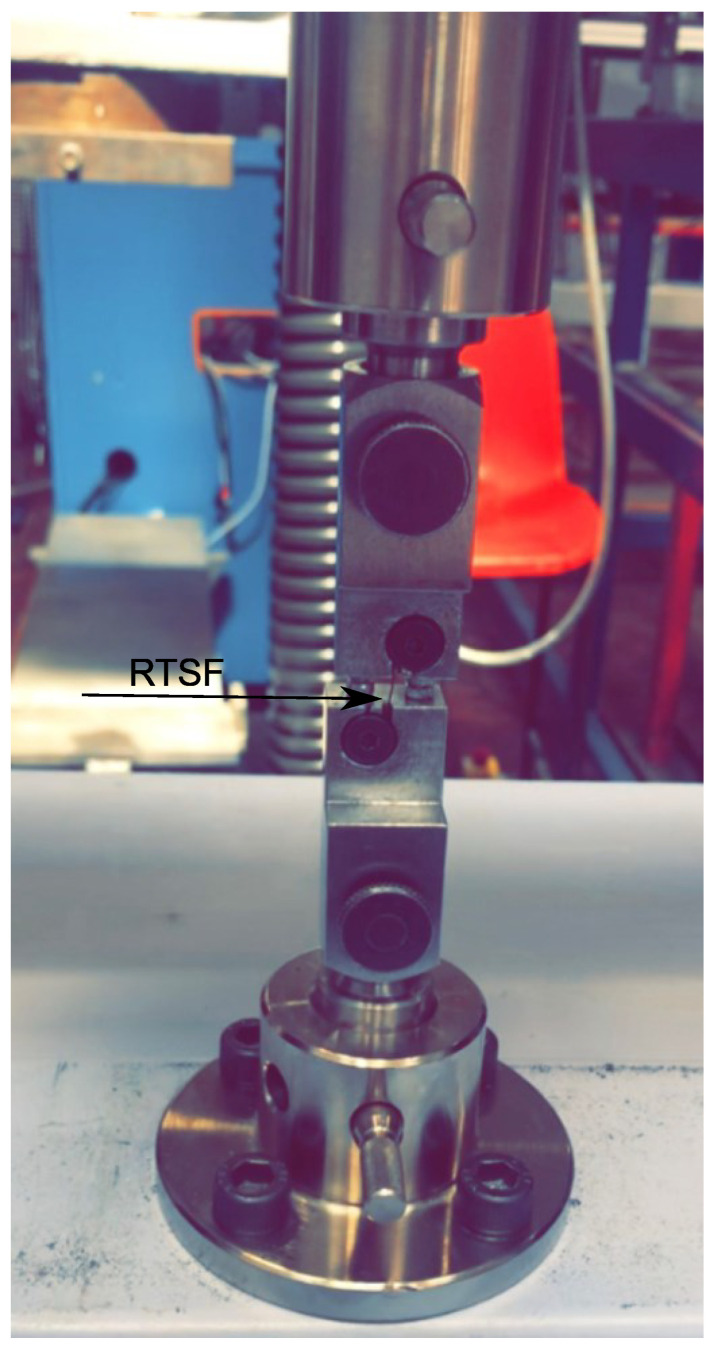
Set-up for tensile testing of RTSF.

**Figure 6:**
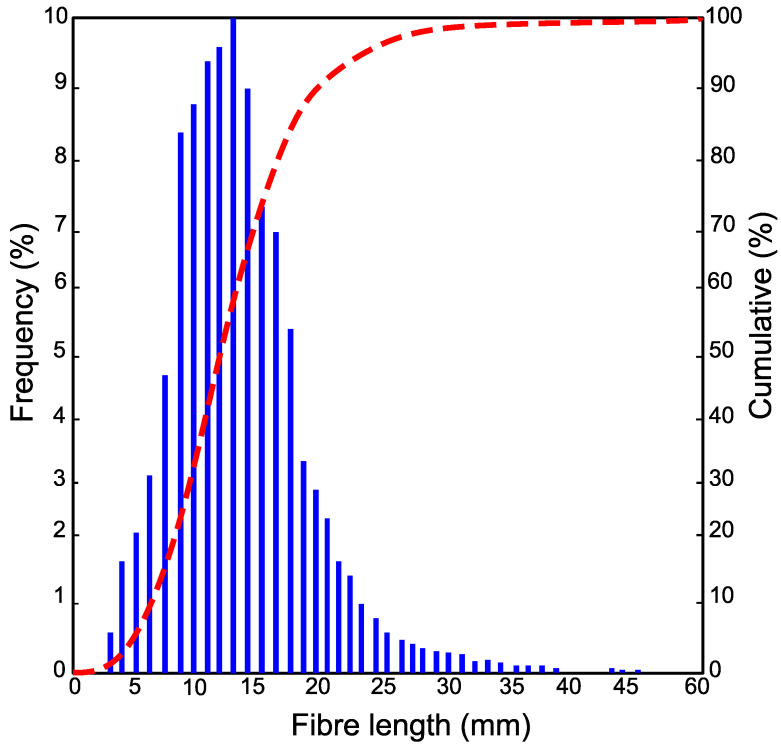
RTSF length distribution.

In this study, specially processed recycled tyre steel fibres (RTSF), as shown in Figure 3, were used in different amounts to assess their performance in controlling plastic shrinkage. The mechanical and physical characteristics of these fibres were obtained by testing more than 100 samples of individual RTSF (see Figure 4). The fibres were tested in tension according to ISO 6892-1 [59] using an electromechanical universal testing machine and specially modified capstan grips, as shown in Figure 5. The average value of the mechanical strength was determined, considering the mechanical performance of the samples that failed within the free length only. Results from samples that failed prematurely at the grips were rejected. The range of fibre lengths used in the experiment was determined by an automated optical method [60] and is shown in Figure 6.

The diameter was measured at 3 points along the fibre length. The average diameter was found to be 0.35 mm (SD = 0.036 mm), with measurements ranging between 0.32 and 0.41 mm (see Figure 4b). The average tensile strength was 2380 Mpa (SD = 166 Mpa), as shown in Figure 4c. Some fibres exceeded 3000 Mpa, which shows that the original cord is of extremely high quality. Nonetheless, the mechanical shredding process used to extract the fibres causes damage, and some fibres fall below 1000 Mpa. However, this loss of strength is unlikely to have any impact on plastic shrinkage cracking control as very little stress develops in the fibres at that stage.

#### 2.1.3. Mix Design

The mix design used in this experimental study is shown in Table 1 and was similar to that used in previous research projects [61]. A higher-than-normal cement content of 335 kg/m^3^ was selected, as this increases the likelihood of plastic shrinkage cracking.

Natural river sand and gravel were used as fine and coarse aggregates, and their total evaporable moisture content was determined by drying, as per [62]. The relative density (specific gravity oven dry (SG_OD_), specific gravity saturated surface dry (SG_SSD_), apparent specific gravity (ASG)) and absorption were also determined, as recommended in ASTM C127-15 [63]. All the properties of fine and coarse aggregates are shown in Table 2. The fine and coarse aggregates were weighed and stored in standard laboratory conditions at 20 ± 2 °C a day before mixing the concrete.

MSF and RTSF were used in different amounts, 0 kg (V_f_ = 0%—control samples), 10 kg (V_f_ = 0.13%), 20 kg (V_f_ = 0.26%), and 30 kg (V_f_ = 0.38%—typical for slabs on grade) per m^3^ of concrete, to examine their impact on plastic shrinkage cracks.

#### 2.1.4. Mixing and Casting Procedure

A pan mixer was used for all mixes and the following mixing/casting procedure was adopted:

1. Materials were weighted according to ASTM C192/C192M [64].
2. Cement and aggregates were dry mixed for 1 min, after which water was added.
3. Mixing continued for 3 min, after which half of the mix was removed to cast the first plastic shrinkage mould (according to ASTM C1579 [53], see next section) and four 100 mm cubes (to measure setting time and compressive strength).
4. The MSF and RTSF were then added and mixing continued for an additional 3 min.
5. The second plastic shrinkage mould was then filled, and four 100 mm cubes were cast.

According to ASTM C1579 [53], the concrete was cast in one layer, followed by a 30 s vibration on a vibrating table. The top surface of the specimen was then screeded three times in the direction perpendicular to the stress riser.

### 2.2. Methodology

#### 2.2.1. Workability

A slump test was carried out in accordance with ASTM C143/C143M [65] to ensure adequate workability after the addition of fibre, with a target of slump of 100 ± 10 mm.

#### 2.2.2. Compressive Strength

The compressive strength of the mixes was obtained in accordance with BS EN, 12390-3 [66] from tests on 100 mm cubes in a servo hydraulic universal testing machine. Two cubes were tested on day 1 to observe the effects of the environmental conditions (air temperature, relative humidity, and airflow) on early-age strength, whilst the other two cubes were tested after 28 days of curing under standard laboratory conditions.

#### 2.2.3. Evaporation Rate

To quantify the base water evaporation rate in the chamber, two aluminium pans were filled with water and were placed next to each slab specimen. The exposed water surface area of each pan was 0.019 m^2^. The evaporation rate at each time interval was determined by (Equation (1)) ASTM C1579 [53]; the mass loss was divided by the surface area of the water and the time interval between successive weighings. The average evaporation rate should exceed 1.0 kg/m^2^/h; otherwise, the test is rejected [53].

(1) $$E=\frac{M2-M1}{water\;saurface\;area\;of\;the\;pan\;\times \left(T2-T1\right)}$$

where *E*: Evaporation rate, kg/m^2^/h; *M*2 − *M*1: the mass loss between successive weighings, g; and *T*2 − *T*1: the time interval between successive weighings, h.

#### 2.2.4. Plastic Shrinkage Test

**Figure 7:**
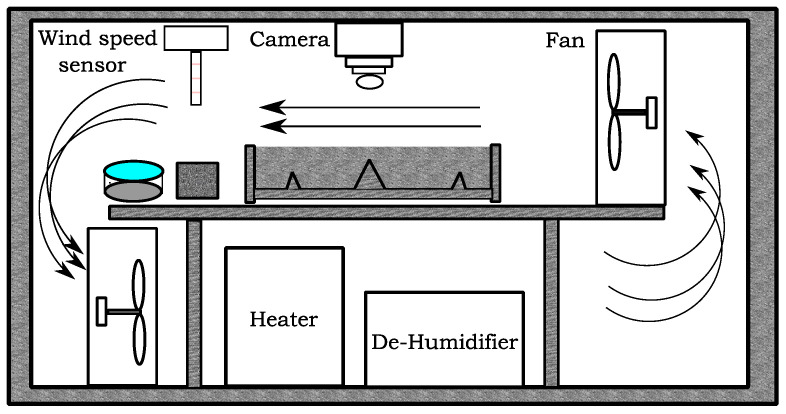
Schematic section of the chamber.

**Figure 8:**
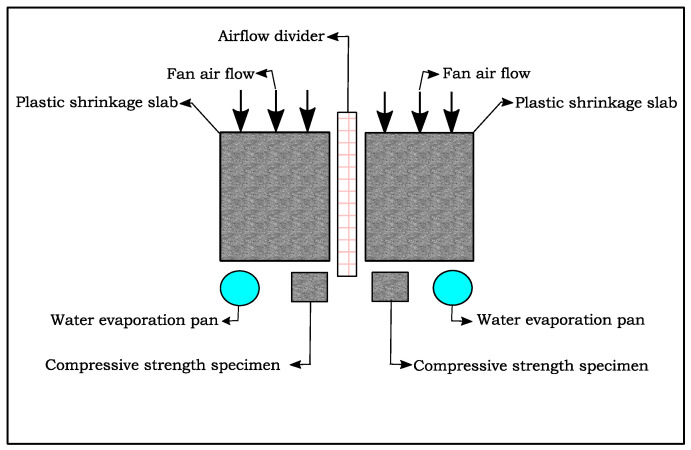
Schematic plan of the contents of the chamber.

Figure 7 and Figure 8 diagrammatically show the chamber ASTM C1579 [53] used to evaluate the performance of different types of fibres in controlling plastic shrinkage cracking. According to ASTM C1579 [53], two comparative specimens were placed in an environmental chamber immediately after casting. The chamber was controlled in terms of air temperature, relative humidity, and airflow. In addition to the specimens, two water pans and two concrete cubes (100 mm) were placed inside the chamber to measure water evaporation and compressive strength of concrete, respectively.

**Figure 9:**
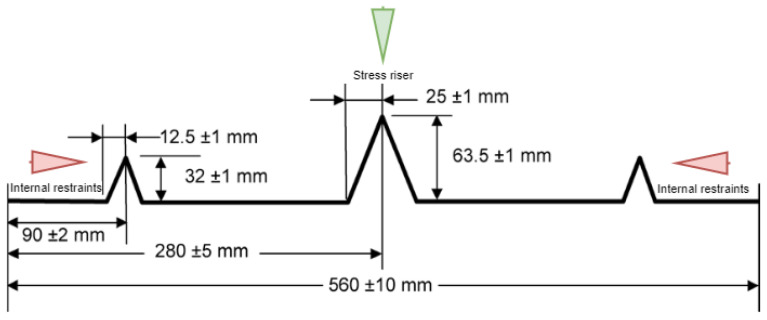
Geometry of the stress riser and internal restraints.

**Figure 10:**
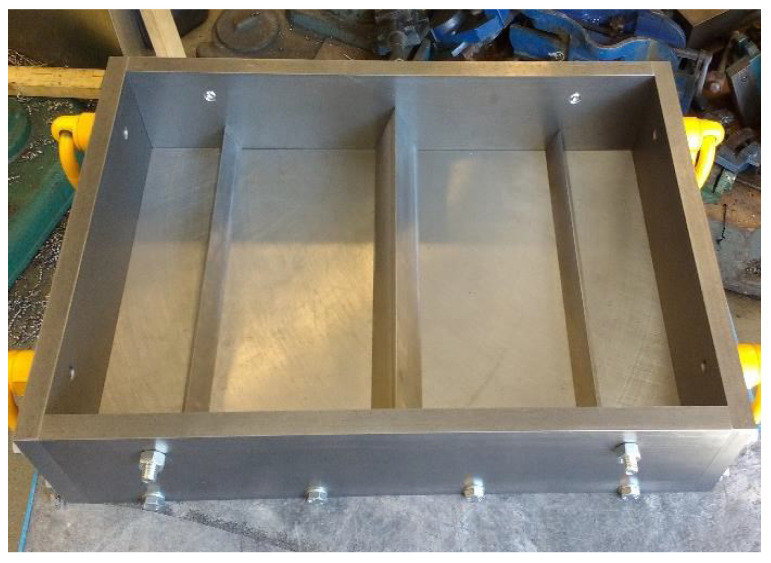
The complete mould.

The plastic shrinkage tests were designed to give a comparative analysis of crack behaviour (width, length, and area) between two slabs in the same environmental conditions. The specimens were rectangular with dimensions of 560 mm in length, 355 mm in width, and 100 mm in depth. A tall triangular stress riser was fixed at the middle of the mould’s steel base to induce cracking, and two shorter triangular internal restraints were fixed close to the sides of the mould (Figure 9) on either side. Both stress risers and internal restraints were made of solid steel. The side and the base of the moulds were made from 20 mm thick steel plates (Figure 10).

The chamber was turned on two hours prior to the start of the test to pre-heat and stabilise the environment to the desired temperature (36 ± 3 °C) and relative humidity (30 ± 10%), as recommended by [53]. Immediately after casting, the moulds and cubes were placed inside the chamber.

**Figure 11:**
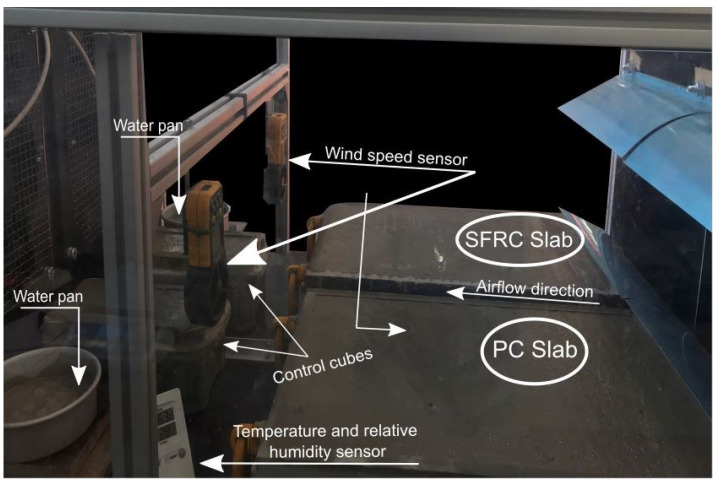
Set-up for plastic shrinkage test.

Records of the water mass loss and water evaporation rate were taken every 30 min, according to ASTM C1579 [53]. The setting time was also monitored every 30 min. The evaporation rate was recorded by removing the monitoring pan from the air stream, weighing it, and returning it to the air stream within 15 s, as recommended in [53]. In addition, the air temperature and relative humidity were recorded every hour, as recommended in [53]. These should be in the range of 36 ± 3 °C and 30 ± 10%, respectively. It should be noted that the wind velocity must be sufficient to maintain the minimum evaporation rate during the test. The wind speed, as recommended in ASTM C1579 [53], should be more than 4.7 m/s over the entire test panel’s surface area. A typical test lasts six hours, after which the fans, heater, and dehumidifier are turned off, the samples covered with plastic sheeting, and the doors of the environmental chamber left open to stabilise to normal lab environmental conditions. Figure 11 shows a photo of specimens in the chamber.

##### Measurements

**Figure 12:**
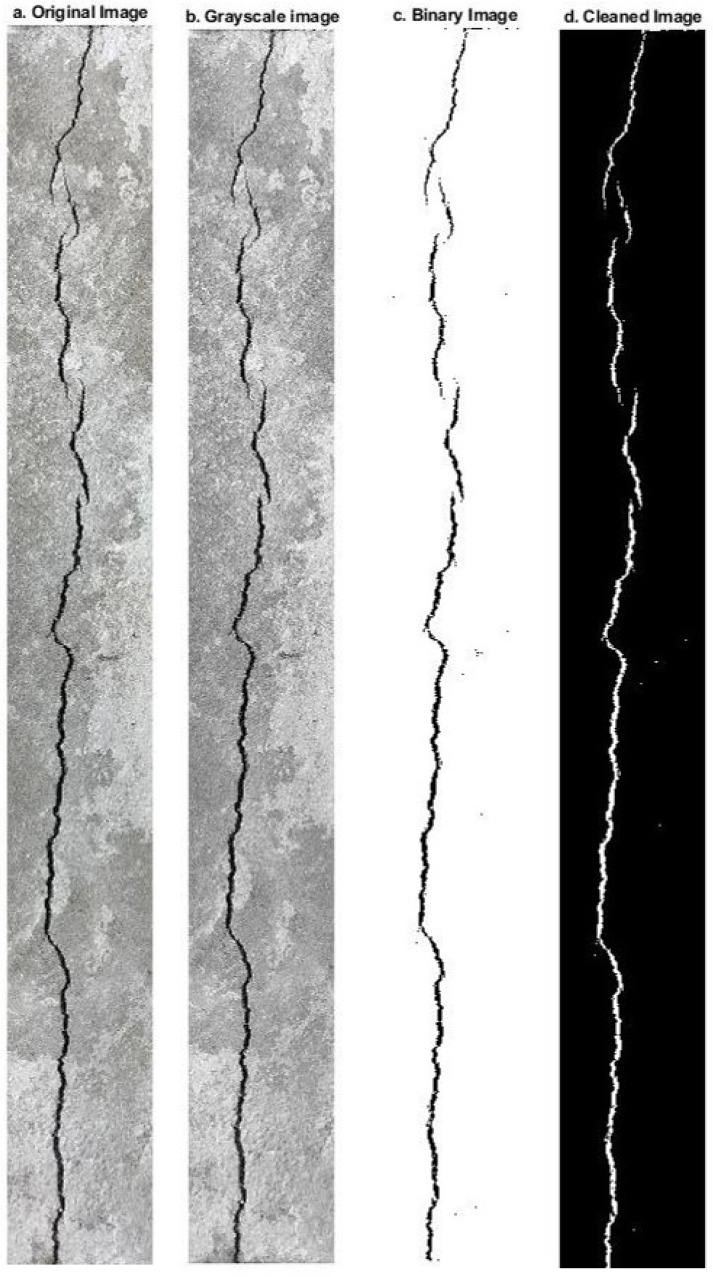
Image processing steps.

Measurements of the plastic shrinkage cracks (length, width, and area) were carried out using a digital image processing method, whereby a camera is placed above the samples during the test to capture images of the samples at different times. The images were then analysed using a digital imaging processing script written in MATLAB. The MATLAB script was written and developed by [67]. The script converts the original images to greyscale (see Figure 12a,b). The greyscale image is then converted to a binary image (see Figure 12c) and cleaned of spurious black pixels (see Figure 12d). This allows the pixels forming the crack to be counted and the average crack width to be determined.

At 24 h, measurements of crack width were obtained by using both digital image analysis, as explained above, and an optical micrometre at more than 25 locations along the crack length to calculate the average of crack width. These measurements were made for comparison purposes between the two methods.

The average plastic shrinkage crack width is used to calculate the crack reduction ratio (CRR) at 6 and 24 h from the start of the test and uses the following equation, as recommended in [53]:

(2) $$\mathrm{CRR}=\left[1-\frac{\mathrm{Average}\;\mathrm{Crack}\;\mathrm{Width}\;\mathrm{of}\;\mathrm{Fibre}\;\mathrm{Reinforced}\;\mathrm{Concrete}\;\mathrm{Mixture}\;}{\mathrm{Average}\;\mathrm{Crack}\;\mathrm{Width}\;\mathrm{of}\;\mathrm{Fibre}\;\mathrm{Control}\;\mathrm{Concrete}\;\mathrm{Mixture}}\right]\times 100\%$$

## 3. Experimental Results and Discussion

### 3.1. Workability

**Figure 13:**
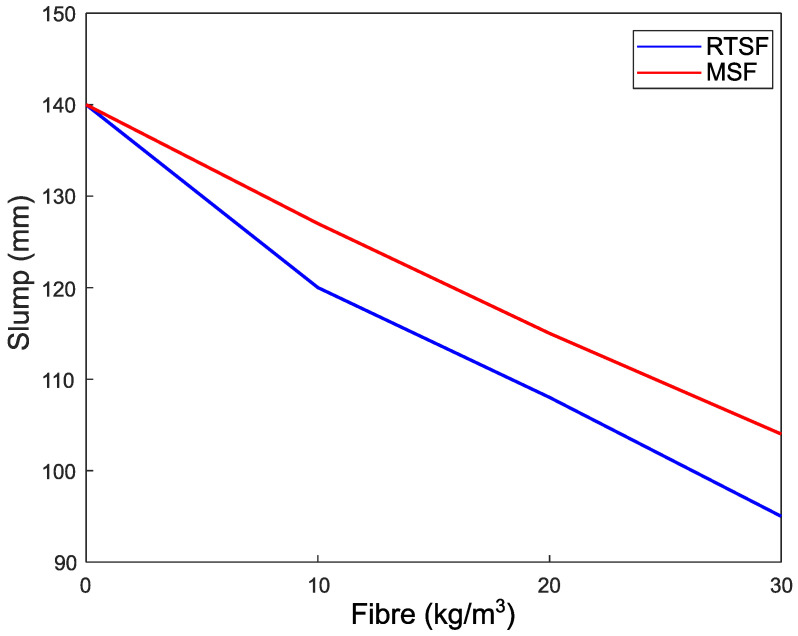
Effect of fibre type and dosage on slump of concrete.

The concrete workability is affected by various factors. It was observed that the addition of RTSF and MSF lead to a reduction in the slump of concrete compared with plain concrete, as shown in Figure 13. This reduction increases with increasing fibre dosage. The RTSF reduced the workability by 20%, 32%, and 45% for doses of 10 kg/m^3^, 20 kg/m^3^, and 30 kg/m^3^, respectively. The corresponding reductions in workability were less when using MSF and were equal to 13%, 25%, and 36%. This is because the number of RTSF fibres is much higher for a single fibre volume compared to MSF, and this also increases the shear resistance of fresh concrete making it harder to flow [68]. These results agree well with published research on RTSF by [26,69].

### 3.2. Compressive Strength

**Figure 14:**
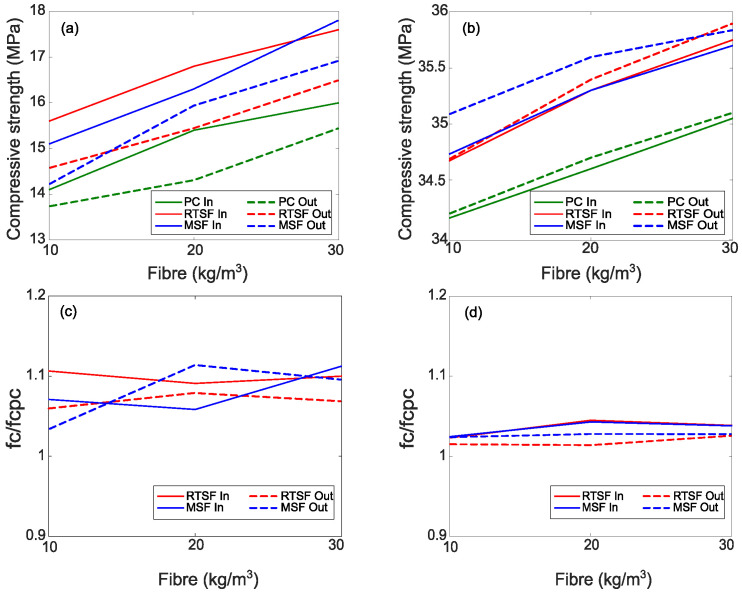
Compressive strength and normalized ratio of PC and FRC at 1 day (**a**,**c**) and 28 days (**b**,**d**) for cubes inside (In) and outside (Out) the chamber.

The compressive strength of control specimens (plain concrete—PC) and steel-fibre-reinforced concrete (RTSF and MSF) at 1 day and 28 days, measured on cubes that were conditioned inside and outside the chamber, are shown in Figure 14, along with the corresponding normalized ratios.

As expected, given the work of [70,71,72], the addition of MSF and RTSF results in only marginal increases in compressive strength, which is seen to increase with fibre content in line with other studies [73,74,75]. Zeybek et al. [76] examined the influence of steel fibres extracted from waste tyres on concrete performance and observed an increase in compressive strength between 17%, 30%, and 46% when adding a volume of fibres from 1%, 2%, and 3%, respectively.

However, the one-day compressive strength after exposure in the chamber for 6 h is around 10% higher than for cubes that were kept outside the chamber (see Figure 14a,c). Dzaye et al. [77] attributes this increase to the higher temperature inside the chamber, which speeds up the hydration reaction. The impact of this early increase in strength is no longer significant after 28 days (see Figure 14b,d).

### 3.3. Evaporation Rate

#### 3.3.1. Environmental Conditions

**Figure 15:**
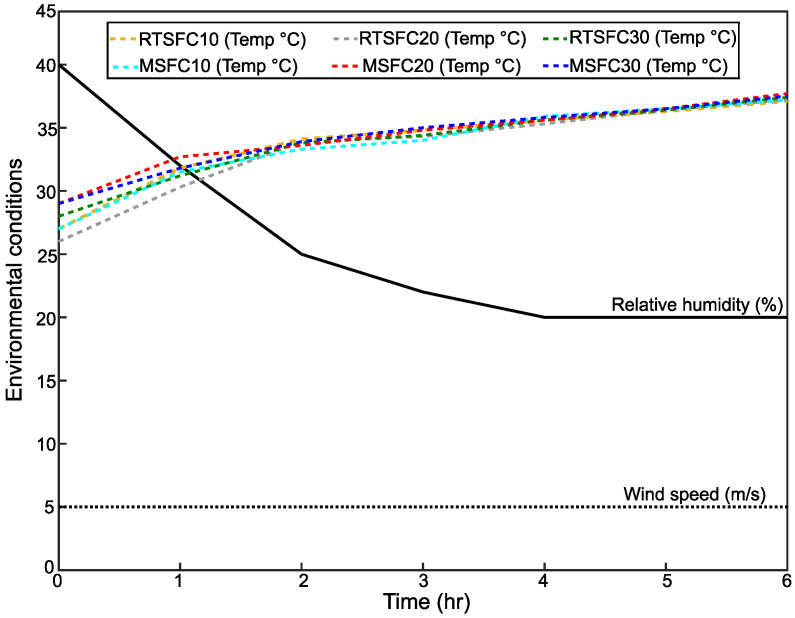
Environmental conditions for all mixes.

The evaporation rate is accepted as one of the main factors that influence the likelihood of plastic shrinkage cracking. When the evaporation rate increases, the crack area and width increases [50,78]. In this study, an attempt was made to expose all test specimens to identical environmental conditions, but the initial material temperature is hard to control precisely due to daily variations in temperatures. Wind speed and relative humidity were controlled, as recommended in ASTM C1579 [53] at 30 ± 10%, and 5 m/s, respectively, as shown in Figure 15. The high drop of relative humidity from 40% to 20% during the test was due to the increase in the air temperature from 28 ± 2 °C to the end of the test at 36 ± 2 °C.

#### 3.3.2. Bleeding and Evaporation Rates

**Figure 16:**
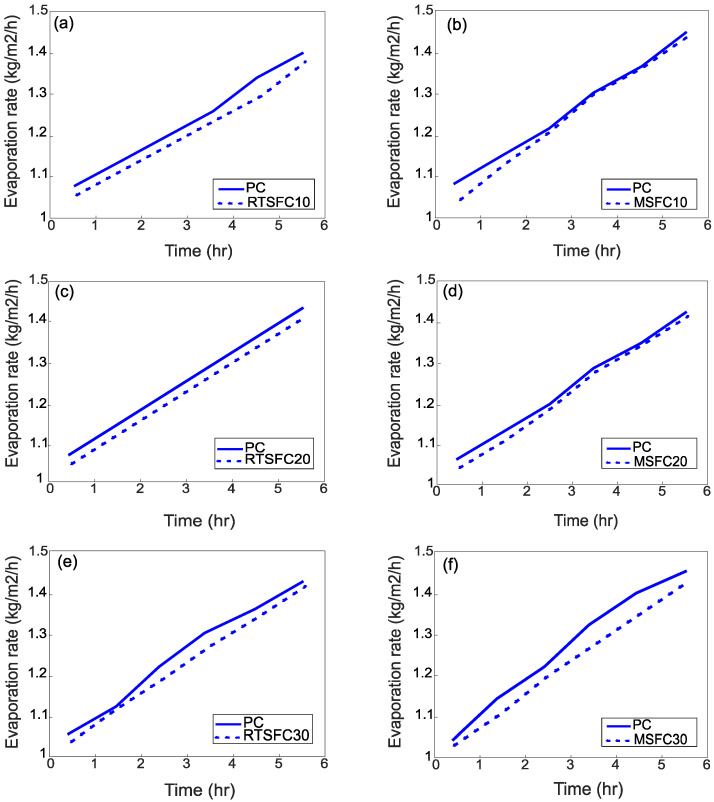
Evaporation rate of the various mixes compared to PC: (**a**) RTSFC10; (**b**) MSFC10; (**c**) RTSFC20; (**d**) MSFC20; (**e**) RTSFC30; (**f**) MSFC30.

Figure 16a–f show the effect of environmental conditions on the evaporation rate of bleed water. All water pan evaporation rates exceed the minimum of 1.0 kg/m^2^/h required by ASTM C1579 [53].

The evaporation rate of all mixes increases roughly in a similar manner, irrespective of fibre amount and type. The increase in the evaporation rate is consistent with the increase in temperature during the test. Initially, the evaporation rate is more or less constant, but after two to three hours of the test the evaporation rate increases slightly. This could indicate an increase in bleed water, but this is unlikely as cracks also appear at this stage, which means that this is likely due to increased surface temperature due to hydration. The development of cracks means that by now the bleeding rate has slowed down or stopped, which is in agreement with other works [79,80].

### 3.4. Plastic Shrinkage Test Results

#### 3.4.1. Study of Cracks on Concrete Surface Using Digital Image Analysis

**Figure 17:**
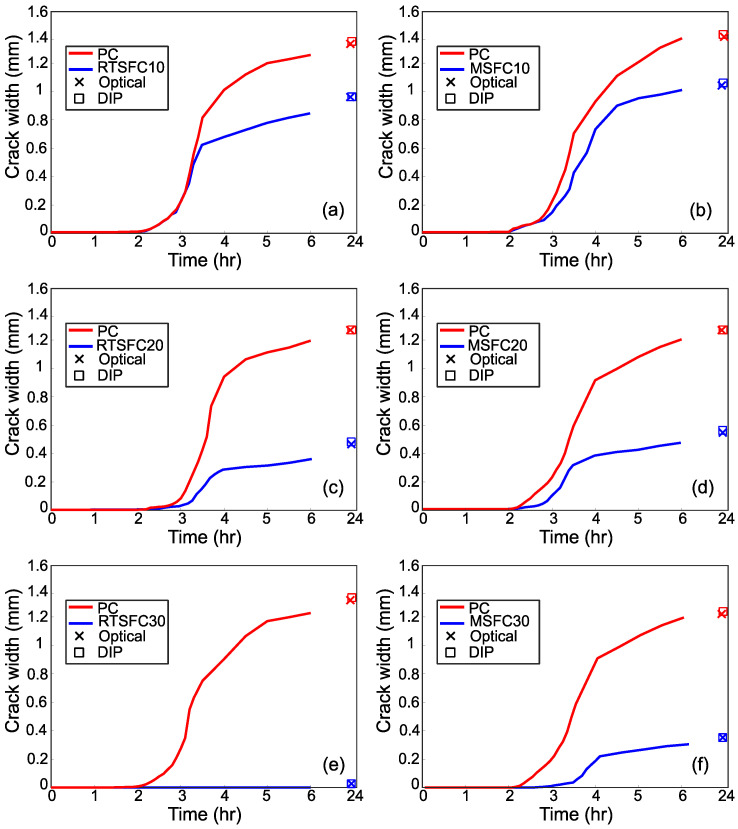
Crack width evolution for all specimens compared to their PC counterpart: (**a**) RTSFC10; (**b**) MSFC10; (**c**) RTSFC20; (**d**) MSFC20; (**e**) RTSFC20; (**f**) MSFC30.

A digital image analysis technique was used to evaluate the evolution of plastic shrinkage cracking during the test. Photos of the concrete surface were taken every 10 min until the cracks first appeared (about two to three hours), and every 30 min thereafter until the end of the test (six hours). Photos were also taken at 24 h, as recommended in [53]. All photos were processed to determine the cracks, as described in Section 3.3.1. The evolution of crack width for all specimens is shown in Figure 17.

Most cracks appear after 2 h, corresponding to the initial setting time, while they show a fast evolution (width) during the first two to three hours after initiation and tend to stabilise towards the end of the test when the concrete is reaching or has reached its final setting time. As expected, after the end of the test and up to 24 h, none of the cracks showed any significant increase in width. These results are in agreement with other similar studies [18,81,82].

The rapid increase in crack width immediately after cracking is attributed to the evaporation rate being equal to or higher than the bleeding rate, which indicates the stage that the concrete surface begins to dry and a negative pore pressure is created [83,84,85]. This negative pore pressure is one of the main causes of surface crack development.

Measurements at 24 h were made using digital image analysis and optical methods for comparison purposes and are also shown in Figure 17. The measurements from the two methods are practically identical, confirming the ability of digital image analysis methods to be used in this application.

#### 3.4.2. Influence of Fibres

Overall, the addition of fibres has a beneficial effect in delaying and preventing plastic cracks, as also reported in [31,86], likely due to their ability to bridge micro-cracks, thus preventing them from joining and propagating.

**Figure 18:**
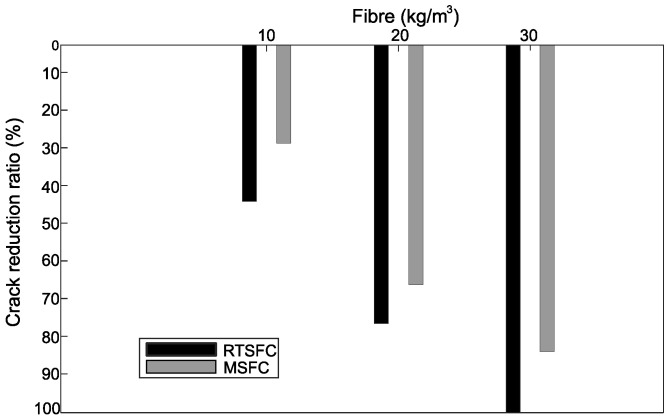
Crack reduction ratio (CRR) in specimens containing RTSF and MSF at 24 h.

**Figure 19:**
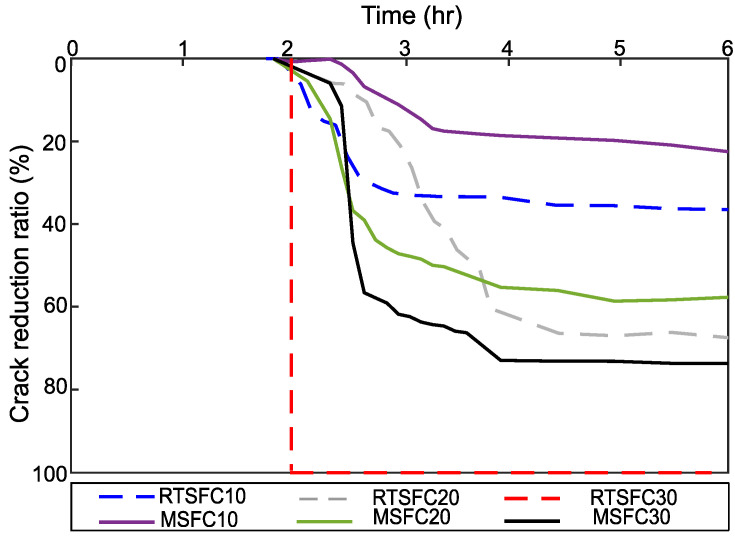
Evolution of crack reduction ratio (CRR) for specimens containing RTSF and MSF at 6 h.

All of the crack reduction ratios (CRR) of this study were determined at the end of the tests, according to Equation (2), and are shown in Figure 18, whilst Figure 19 shows the evolution of CRR with time.

Both fibre types (RTSF and MSF) show a good performance in controlling plastic shrinkage cracking. Better crack control is achieved at increasing fibre dosages, and cracking is avoided completely when using 30 kg/m^3^ of RTSF. This was also observed in [87].

#### 3.4.3. MSF vs. RTSF

**Figure 20:**
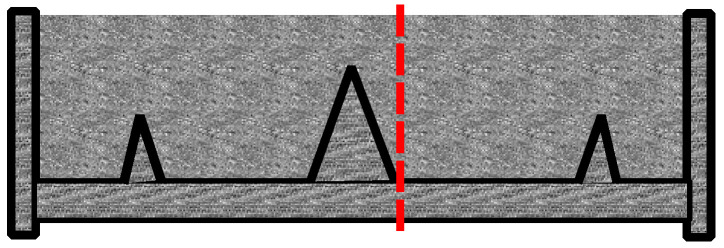
Location of the cross-section of RTSF and MSF slabs (red dashed line).

**Figure 21:**
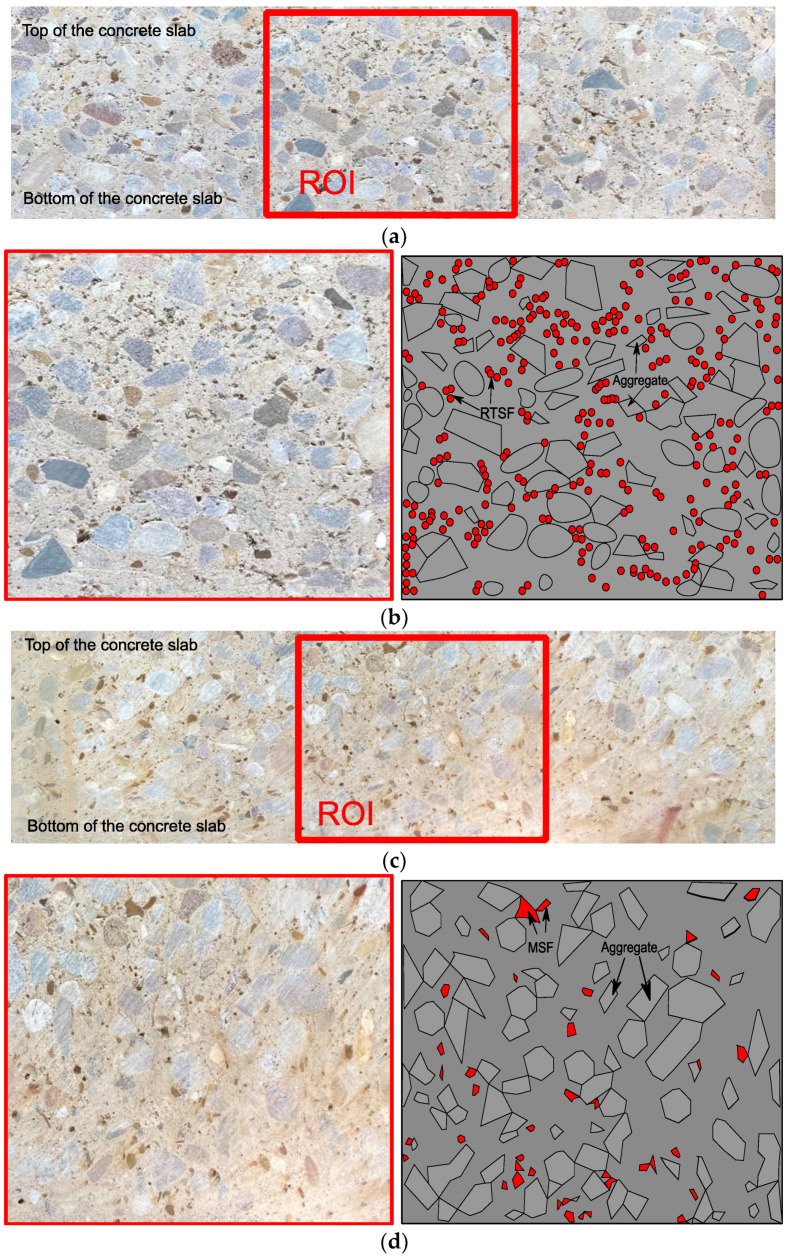
(**a**) Cross-section of specimen RTSFC30 and selected region of interest (ROI). (**b**). Magnified ROI of cross-section of specimen RTSFC30 (**left**) and distribution of aggregates and fibres (**right**). (**c**). Cross-section of specimen MSFC30 and selected region of interest (ROI). (**d**). Magnified ROI of cross-section of specimen MSFC30 (**left**) and distribution of aggregates and fibres (**right**).

The impact of RTSF on plastic shrinkage cracking has not been discussed in previous studies. Overall, the results show that RTSF outperforms MSF at all dosages, and the qualitative image analysis presented below seems to confirm that the number of fibres, their weight, and their overall rigidity play a role in their performance. Cross-sections of the RTSF and MSF slabs were cut in the proximity of the stress riser, as shown in Figure 20. These sections are examined for fibre distribution, as shown in Figure 21a–d. Overall, RTSF appear to be better distributed within the mix (Figure 21c) than the MSF (Figure 21d). This can be attributed to the smaller diameter of the RTSF, which results, for the same dosage, in a much larger number of fibres that are available to reinforce the matrix and to intersect possible cracks. Furthermore, their larger surface area relative to their weight means that they are less likely to sink to the bottom than MSF (see Figure 21d). Finally, the irregular shape and flexibility of RTSF means that they can more easily move around aggregates and fill gaps, rather than control and restrain the distribution of the aggregates via their rigidity.

## 4. Conclusions

This paper investigates the effect of different dosages of RTSF and MSF on restraining concrete plastic shrinkage and micro cracks at the fresh stage. Plain concrete and fibre-reinforced concrete slab specimens are tested according to the test method recommended in ASTM C1579 under controlled environmental conditions, and their crack initiation and development is examined, along with other physical parameters. From the analysis of the results presented in this paper, the following conclusions can be drawn:

- The evaporation rates are similar for all specimens and increase with increasing temperature.
- Cracking initiates after approximately 2 h from casting, indicating the initial setting time, and substantially stopped after 6 h, which can be considered as the final setting time.
- Exposure to the higher temperature in the chamber increases the hydration rate and 24 h strength of the concrete, although this had no major impact on the 28-day strength. The fibres only have a minor enhancing effect on compressive strength.
- RTSF outperform MSF in mitigating plastic shrinkage cracking at all dosages, with CRR values of 42%, 75%, and 100% for fibre dosages of 10 kg/m^3^ (V_f_ = 0.13%), 20 kg/m^3^ (V_f_ = 0.26%), and 30 kg/m^3^ (V_f_ = 0.38%), respectively. The better performance of RTSF is attributed to their larger number and better distribution within the concrete volume when compared to MSF.

Although an appropriate fibre volume should be selected depending on mix design and target overall performance, a fibre dosage of 30 kg/m^3^ (V_f_ = 0.38%) can be used to prevent plastic shrinkage cracking completely in most typical applications and environmental conditions.

This study provides compelling experimental evidence that RTSF are a sustainable and effective alternative to MSF in preventing plastic shrinkage cracks, in the same way as they are effective against drying shrinkage. The use of finer fibres has a beneficial effect in controlling plastic shrinkage cracks and their use (or the use of blends of MSF and RTSF) is expected to provide structural, durability, and sustainability benefits, particularly in the harsher environmental conditions created by climate change.

Future studies should examine the synergistic effects of using sustainable fibre alternatives, such as the RTSF used in the work presented in this paper, with different cement and aggregate replacements, as well as different curing methods to reduce or prevent plastic shrinkage cracking in concrete.

## Figures and Tables

**Table 1:** Concrete mix proportions.

Material	Quantity
Cement (CEMII 42.5)	335 kg/m^3^
Fine Aggregate (dry) (river round sand)	847 kg/m^3^
Gravel 10 mm (dry) (river round gravel)	491 kg/m^3^
Gravel 14 mm (dry) (river round gravel)	532 kg/m^3^
Water	185 kg/m^3^
Superplasticiser (Twinflow) (Sika ViscoCrete 30HE)	1.5 lt/m^3^
MSF	10 kg/m^3^, 20 kg/m^3^, and 30 kg/m^3^
RTSF	10 kg/m^3^, 20 kg/m^3^, and 30 kg/m^3^

**Table 2:** Physical properties of fine and coarse aggregates.

Bulk Density of Aggregate	Fine Aggregates	Coarse Aggregates Size 10 mm	Coarse Aggregates Size 20 mm
Moisture %	2.58	0.83	0.24
SG_OD_	3.18	2.50	2.60
SG_SSD_	3.21	2.50	2.60
ASG	3.30	2.58	2.66
Absorption%	1.23	0.91	0.58

Note: SG_OD_ = specific gravity oven dry; SG_SSD_ = specific gravity saturated surface dry, ASG = apparent specific gravity.

## Data Availability

Not applicable.
